# Visitor Attitudes Toward Little Penguins (*Eudyptula minor*) at Two Australian Zoos

**DOI:** 10.3389/fpsyg.2021.626185

**Published:** 2021-02-11

**Authors:** Samantha J. Chiew, Paul H. Hemsworth, Vicky Melfi, Sally L. Sherwen, Alicia Burns, Grahame J. Coleman

**Affiliations:** ^1^Faculty of Veterinary and Agricultural Sciences, Animal Welfare Science Centre, University of Melbourne, Parkville, VIC, Australia; ^2^Animal & Agriculture Research Centre, Hartpury University, Gloucester, United Kingdom; ^3^Department of Wildlife Conservation and Science, Zoos Victoria, Parkville, VIC, Australia; ^4^Taronga Institute of Science and Learning, Taronga Conservation Society Australia, Mosman, NSW, Australia; ^5^School of Life and Environmental Sciences, University of Sydney, Sydney, NSW, Australia

**Keywords:** comparisons between zoos, little penguins, visitor attitudes, visitor experience, zoos, zoo visitors, visitor-animal interactions

## Abstract

This study identified and compared the attitudes of visitors toward zoo-housed little penguins, their enclosure and visitor experience that may influence the way visitors behave toward little penguins at two Australian zoos. Visitor attitudes were assessed using an anonymous questionnaire, targeting visitor beliefs, and experiences, where visitors were randomly approached at the penguin exhibit after they had finished viewing the penguins. Visitors were given two options to complete the questionnaire, on an iPad during their zoo visit or online (URL sent via email) after their zoo visit. A total of 638 participants (495 at Melbourne Zoo and 143 at Taronga Zoo) completed the questionnaire, 42% were completed onsite during their zoo visit and 58% were completed online after their zoo visit. Most participants were living in Australia, non-zoo members, female, previously or currently owned a pet, aged between 26 and 35 years and had a University degree. Results showed that the attitude dimensions of visitors were consistent between the two zoos which indicates that these measures of attitudes were stable over time and location. Overall, visitors at both zoos had positive attitudes toward little penguins, penguin welfare, the enclosure, and visitor experience. However, whether these positive attitudes and positive visitor experience influenced the way visitors behaved toward the penguins remains unclear. There were some differences in visitor attitudes toward the perceived “aggressiveness” and “timidness” of little penguins, “negative penguin welfare”, “experience with the penguins”, “learning”, “visual barriers” and the way visitors rated their overall experience at the penguin enclosure. While the reasons for the differences in visitor attitudes and visitor experience between the zoos were not clear, some factors such as penguin behavior and enclosure design, may have been attributable to these differences. Also, a relationship was found between visitor attitudes and how visitors rated the welfare of penguins, the enclosure and visitor experience at the enclosure; more positive visitor attitudes were associated with higher ratings of penguin welfare, the enclosure and visitor experience. The practical implications of these results for zoos is unclear because the differences in visitor attitudes were numerically small. This requires further comparisons between zoos or enclosures that are more markedly different than the penguin enclosures in the present study and further research on how visitors assess zoo animals, enclosures and visitor experience.

## Introduction

Visitors are important targets of many zoo conservation, education, and advocacy campaigns that are designed to mitigate human-driven threats to wildlife by influencing visitor attitudes and behavior (Reade and Waran, 1996; Davey, 2007; Skibins and Powell, 2013; Godinez and Fernandez, 2019; Howell et al., 2019). This is important because conservation and education forms the ethical foundation and justification of zoos for keeping wildlife in captivity (Maple, 2008; Ward and Sherwen, 2018; Godinez and Fernandez, 2019; Sherwen and Hemsworth, 2019). But a number of factors appear to influence visitor attitudes toward zoos and their animals, conservation-related attitudes and the behavior of zoo visitors. For example, some of these factors include enclosure design, visitor interactions with animals, animal behavior as well as the frequency of zoo visits (Woods, 2002; Yalowitz, 2004; Lukas and Ross, 2005; Miller et al., 2013; Clayton et al., 2017; Luebke, 2018; Chiew et al., 2019b; Godinez and Fernandez, 2019). Nevertheless, knowledge of visitor attitudes is important because attitudes are drivers of behavior (Ajzen, 1991). By understanding visitor attitudes, zoos can gain a better understanding of their visitors and their behavior which can be targeted to promote visitor support for zoos, proconservation behaviors and raise visitor awareness of wildlife conservation (Lukas and Ross, 2005; Martín-López et al., 2007; Pearson et al., 2013, 2014a,b; Mellish et al., 2019). It is important that we define what “attitudes” are because they are often interchangeably used with “perceptions.” Attitudes are a complex combination of human factors including personality, beliefs, values, behaviors, and motivations (Ajzen, 1985; Pickens, 2005). However, they can be defined by three interconnected components: a person's beliefs about the object (cognition), their emotional response to the object (affect), and their behavioral tendency toward the object (conation) (Ajzen, 1985; Pickens, 2005; Hemsworth and Coleman, 2011; Albarracin et al., 2014). Therefore, attitudes are the mindset or tendency for a person to act in a certain way and reflect a positive or negative assessment of a given object, which are derived from beliefs and are a strong determinant of behavior (Ajzen, 1985; Eagly and Chaiken, 1993; Pickens, 2005). Perceptions, in comparison, are an individual's interpretation of given situations, stimuli, or objects into something meaningful based on past experiences (Ajzen, 1985; Pickens, 2005).

Much of the research to date examining zoo visitor attitudes has primarily been focused on evaluating the impact of zoo-based conservation education and the zoo experience on visitors' knowledge of and attitudes toward zoo animals (Falk et al., 2007; Marino et al., 2010; Miller et al., 2013; Pearson et al., 2013, 2014a,b; Roe et al., 2014; Godinez and Fernandez, 2019; Mellish et al., 2019; Kleespies et al., 2020). These aspects of visitors, knowledge and attitudes, may be related to a visitor's behavioral intention to engage in proconservation-related behaviors (Lukas and Ross, 2005; Martín-López et al., 2007; Godinez and Fernandez, 2019). For example, while not a linear relationship, zoo visitors who were found to have greater knowledge about orangutans (*Pongo abelii*) and dolphins, had more positive attitudes toward these animals which in turn, were found to be associated with increased behavioral intentions of engaging in future conservation behavior for these species (Miller et al., 2013; Pearson et al., 2013, 2014a). In comparison, more potentially harmful behaviors toward marine wildlife and less engagement in proconservation behaviors were found when public attitudes toward marine wildlife were negative and knowledge of marine wildlife and their related conservation issues were poor (Barney et al., 2005; Pearson et al., 2014b). This relationship between knowledge, attitudes and behavior is based on the Theory of Planned Behavior which proposes that human behavior may be predicted by attitudes toward their behavior, subjective norms and the perceived behavioral control of their behavior (Ajzen, 1991; Fishbein and Ajzen, 2010). Consequently, evaluation of the zoo experience on zoo visitors is important because it helps identify the effectiveness of zoos in promoting behavioral change in their visitors for the purpose of wildlife conservation and education (Whitham and Wielebnowski, 2013; Roe et al., 2014; Sherwen et al., 2018; Godinez and Fernandez, 2019). However, little is known about whether the attitudes of visitors toward zoo animals and visitor experience influences the way visitors behave toward zoo animals.

In situations where there is close proximity between stockpeople and their livestock, attitudes toward livestock can influence the way stockpeople behave toward animals they work with (Hemsworth and Coleman, 2011). The relevant stockperson behavior includes not only tactile contact, but also other aversive behaviors including speed of movement and noise production. Positive attitudes in stockpeople toward livestock and working with livestock has been shown to decrease aversive behaviors and result in positive outcomes on animal behavior and welfare (Hemsworth and Coleman, 2011; Munoz et al., 2019). Whether similar relationships between zoo visitor attitudes and behaviors that may be aversive to zoo animals remains poorly understood. There has been a substantial increase in evidence that zoo visitors can affect a wide range of zoo species (Fernandez et al., 2009; Ward and Sherwen, 2018; Sherwen and Hemsworth, 2019). Most research to date has found that zoo visitors can negatively affect many zoo species, shown by increases in animal behaviors such as aggression, avoidance, and stereotypies or increases in physiological measures such as fecal glucocorticoid metabolite concentrations (Sherwen and Hemsworth, 2019). Despite these findings, there is limited understanding of what visitor behaviors result in these negative effects on zoo animals. There is evidence to suggest that it may be certain visitor behaviors that are generally fear-provoking, particularly those that are intense, loud, fast, and sudden, that negatively affect zoo animals (Hemsworth et al., 2018; Sherwen and Hemsworth, 2019). Therefore, potentially fear-provoking visitor behaviors such as looming over animals, loud vocalizations, or sudden movements may be influenced by visitor attitudes toward zoo animals and the experience of visitors while at the zoo or specific animal exhibit. For example, visitor perceptions of the gorillas (*Gorilla gorilla gorilla*) were perceived as more exciting and less aggressive when camouflage netting was installed at the viewing area of the exhibit (Blaney and Wells, 2004). At the same time, the camouflage netting reduced conspecific-directed aggression and stereotypic behavior in gorillas (Blaney and Wells, 2004). It is apparent that visitor perceptions of the gorillas were influenced by the camouflage netting and the change in gorilla behavior. But, there was some suggestion that visitors became quieter and engaged in less intense behaviors (e.g., banging on the glass windows) toward the gorillas when the netting was installed (Blaney and Wells, 2004). The change in visitor behavior may have also been influenced by their improved perceptions of the gorillas although association specifically between visitor perceptions of gorillas and visitor behavior was not examined. Furthermore, visitor experiences at zoos such as presentations, informative signage, immersive naturalistic exhibits and interactions with zoo animals and staff, have been found to influence visitor attitudes toward zoo animals and visitors' emotional connections with and appreciation for zoo animals and learning (Reade and Waran, 1996; Miller et al., 2013; Mann et al., 2018; Godinez and Fernandez, 2019). Consequently, understanding visitor attitudes toward zoo animals and their connection with visitor experience can provide valuable insight into the attitudes that may drive the way visitors behave toward zoo animals. These attitudes can then be targeted by zoos to promote behavioral change in visitors to help zoos better manage visitor-animal interactions.

Penguins are commonly housed in zoos and have been referred to as a charismatic species based on their aesthetic appeal, being perceived as “cute” and have the ability to invoke a large amount of emotional affinity (Woods, 2000; Stokes, 2007; Tucker, 2007; Seddon and Ellenberg, 2008; Colléony et al., 2017; Chiew et al., 2020). Yet, there has been limited research to understand the attitudes of visitors toward this group of aquatic birds and lack of bird species being studied in general (Hosey et al., 2020). Additionally, there has been a lack of research comparing visitor attitudes and visitor experience between zoos. Some studies that have investigated the affective responses of visitors to observing animal behavior have involved a range of zoos in order to capture a wide range of visitors and account for the variation in visitor responses between zoos (Myers et al., 2004; Luebke et al., 2016; Godinez and Fernandez, 2019). However, research to date has yet to focus on understanding the differences and/or similarities in visitor attitudes toward a specific zoo species and visitor experience between zoos. Visitor attitudes toward the same zoo species at different zoos may be the same while visitor experience could differ between zoos because of factors such as features within zoos that promote positive experiences. By comparing visitor attitudes toward specific zoo species and visitor experience between zoos, this can help identify factors that may influence visitor attitudes and experiences that generalize across zoos.

The present study targeted visitors' beliefs about the little penguins, their enclosure and visitor experience. This is because beliefs, as one of the three components of attitudes, permits an individual's underlying attitudes to be inferred (Eagly and Chaiken, 1993; Pickens, 2005; Hemsworth and Coleman, 2011; Albarracin et al., 2014). Consequently, the term “attitude” as used in this study refers to visitor beliefs. The aim of the present study was to identify and compare the attitudes of visitors toward zoo-housed little penguins, their enclosure and visitor experience that may influence the way visitors behave toward little penguins at two Australian zoos.

## Methodology

### Visitor Questionnaires

This study obtained human ethics approval from the Veterinary and Agricultural Sciences Human Ethics Advisory Group, Faculty of Veterinary and Agricultural Sciences, University of Melbourne, Australia (Ethics Application 1545739.1 and 1545739.3). Questionnaires were developed and refined based on focus groups discussions with visitors at Melbourne Zoo (Melbourne, Australia) and Taronga Zoo (Sydney, Australia) (Chiew et al., 2019b). A slightly modified version of the questionnaire used in Chiew et al. (2019b) was used at Taronga Zoo and implemented a similar methodology. Only question items that were consistent across the two zoos were included in this present study.

Visitors at both zoos were randomly approached at the penguin exhibit after they had finished viewing the penguins and had exited the exhibit area by a member of the research team (student volunteers, interns or a research assistant from the Animal Welfare Science Centre, University of Melbourne). At Melbourne zoo, visitors were approached during seven 30 min time blocks between 09:30 and 15:15 h (Chiew et al., 2019b). At Taronga zoo visitors were approached between 11:00–12:00 h and 14:00–15:00 h. For Melbourne Zoo, the time blocks were selected based on visitors being found to visit the penguin enclosure throughout the day. At Taronga Zoo, the selected times blocks were identified to be the peak times visitors were located at the penguin exhibit area. To increase participation, visitors were provided two options to complete the questionnaire, either on an iPad (on site during their zoo visit) or online (URL sent via email, after their zoo visit).

The questionnaire took no longer than 10 min and was divided into five sections: Section 1 collected information on the participants' demographic information; Section 2 collected information on the participants' attitudes toward the little penguins; Section 3 collected information on the participants' attitudes toward the little penguin enclosure, Section 4 collected information on the participants' experience; and the final section assessed the participants' attitudes toward manipulations to the penguin enclosure.

Questionnaires were conducted on all study days during zoo opening hours. Attitude questions within the questionnaire used a 5-point Likert scale with the following options: 1. Strongly disagree, 2. Disagree, 3. Neither agree nor disagree, 4. Agree, and 5. Strongly agree. Responses were scored so that disagreement with a statement had lower scores and agreement had higher scores. For rating questions in which visitors were asked to rate the welfare of the little penguins, the little penguin enclosure and visitor experience at the penguin enclosure, a rating scale out of 10 was utilized where 1 was very poor and 10 was excellent.

Visitor attitudes were surveyed in conjunction with our studies that investigated the effects of regulating visitor contact on little penguin behavior at Melbourne and Taronga Zoo, using exhibit manipulations: a physical barrier and visual barrier, respectively (Chiew et al., 2019a, 2020). Consequently, the number of days questionnaires were collected differed between the zoos as a result of the differing experimental designs i.e., 24 study days at Melbourne Zoo and 10 study days at Taronga Zoo.

### Data Analysis

Statistical analyses using SPSS version 25 included frequency distributions of demographic factors across response categories in the questionnaire and principal component analyses (PCAs) on attitudinal data at each zoo. Before PCAs were performed, any questions that were not consistent across the two zoos were removed to ensure only questions that were the same at the two zoos were included for analysis. Attitudinal data from the questionnaire were subjected to PCAs for each zoo separately, to reduce the large number of attitude variables to a relatively small number of components, where the components reflected commonalties amongst those individual variables that correlated highly with each other. The Kaiser-Meyer-Olkin Measure of Sampling Adequacy and Bartlett's test of sphericity values were used to assess the suitability of the data set to be subjected to PCA (Pallant, 2007). The criterion for the Kaiser-Meyer-Olkin Measure of Sampling Adequacy is 0.60 or above and Bartlett's test of sphericity had to be significant (*p* < 0.05) which indicated the data set was suitable for PCA (Pallant, 2007). Also, using Kaiser's criterion, only components with eigenvalues exceeding 1 were retained (Pallant, 2007). Cronbach's α coefficients were used as a measure of scale reliabilities, with an α ≥ 0.70 as the criterion for acceptable reliability (Pallant, 2007). Scale reliability is the degree to which the question items, that make up the component, are assessing the same underlying construct (Briggs and Cheek, 1986; Pallant, 2007). When Cronbach's α coefficients were found to be below the criterion, inter-item correlations were used as a secondary measure of scale reliability as low Cronbach's α can be the result of a scale containing fewer than 10 items. Consequently, inter-item correlations were used when this situation was encountered and the criterion for acceptable reliability is within the range of 0.20–0.50 (Briggs and Cheek, 1986).

Items were included in a component if their loading on the relevant component exceeded 0.33 and if, based on face validity and semantic content, they could be summarized by a single construct (Pallant, 2007; Tabachnick and Fidell, 2007). Varimax or oblimin rotations were performed on component solutions of more than one factor to simplify interpretation (Pallant, 2007). The decision about which rotation was used was based on the component correlation matrix. If correlations were greater than 0.30 within this matrix, indicating that there was more than 10% overlap in variance between the components and so were considered correlated, an oblimin rotation was used (Pallant, 2007; Brown, 2009). If correlations were below 0.30, then a varimax rotation was used. Loadings above 0.70 were considered strong/excellent (Tabachnick and Fidell, 2007). Subjective labeling of each component, produced from the PCAs, based on semantic content of the items was performed. Scale mean scores for each component were then calculated so that the averages were on the same scale as the original question items i.e., 5-point Likert scale. Individual questions related to little penguin aggressiveness, timidness, and cuteness (i.e., do you think little penguins are aggressive/timid/cute?) were analyzed separately because these question items did not reliably measure a common underlying construct when subjected to PCA. Also, the three rating questions where visitors were asked to rate the welfare of the little penguins, the enclosure and visitor experience at the enclosure were analyzed separately because a different rating scale (i.e., out of 10) to the attitudinal statements was used. Therefore, a total of six individual question items were analyzed separately as single items in addition to the scale mean scores of the PCA components.

From each zoo, the scale mean scores for each component and the individual question items were used as measures of visitor attitudes for subsequent statistical analyses. Chi square tests were conducted to identify differences in visitor demographics between the two zoos. Multivariate analysis of variance (MANOVA) on the scale mean scores for each component and the individual question items were conducted to make comparisons of visitor attitudes between the two zoos, demographics and the interaction between zoo property and demographics. Prior to these MANOVAs, Levene's test statistic was used to test for homogeneity of variance. When differences in visitor attitudes (i.e., dependent variables) between the zoos and/or demographics (i.e., independent variables) were identified from MANOVAs, univariate analyses were then examined to identify which attitudes differed between the two zoos and/or demographics. Partial correlations, which were controlled for zoo property, were used to examine the relationship between visitor attitudes and how visitors rated little penguin welfare, the penguin enclosure and visitor experience.

## Results

### Demographics

A total of 638 participants completed the questionnaire, 271 were completed onsite during their zoo visit (42%; 238 at Melbourne Zoo and 33 at Taronga Zoo) while 367 were completed online after their zoo visit (58%; 257 at Melbourne Zoo and 110 at Taronga Zoo). At both zoos, most participants that completed the questionnaire were living in Australia, non-zoo members, female, were previously or currently pet owners, aged between 26 and 35 and had a University degree (Table 1). However, chi square tests (χ^2^) found there were some differences in visitor demographics between the two zoos including “Residence,” “Type of visitor,” “Pet ownership,” and “Age.” At Melbourne Zoo, a greater proportion of visitors living in Australia completed the questionnaire while at Taronga Zoo nearly an equal number of both local and international visitors completed the questionnaire (χ^2^ = 30.21, *n* = 633, *p* < 0.01, phi = 0.22; Table 1). Also, there was nearly a 50/50 split of zoo members and non-zoo members at Melbourne Zoo that completed the survey while more non-zoo members completed the questionnaire at Taronga Zoo (χ^2^ = 45.94, *n* = 637, *p* < 0.01, phi = 0.27; Table 1). A greater proportion of pet owners at Melbourne Zoo compared to Taronga Zoo completed the questionnaire despite at both zoos, visitors that had previously or currently owned a pet primarily completed the survey more than non-pet owners (χ^2^ = 10.02, *n* = 637, *p* < 0.01, phi = 0.13; Table 1). Also, even though most participants at both zoos were aged between 26 and 35 years of age, there was a greater number of participants that completed the questionnaire at Melbourne Zoo aged between 26–35 and 36–45 compared to Taronga Zoo where participants were primarily aged between 18–25 and 26–35 (χ^2^ = 11.12, *n* = 634, *p* < 0.05, Cramer's V = 0.13; Table 1). A chi square test was also performed on “Education” which was not significant (*p* > 0.05) but it should be noted this was performed with the categories *No formal schooling, Primary school*, and *Other education institution* removed as these categories were found to have frequencies less than 5.

**Table 1:** Comparison of demographic information of participants between the two zoos using Chi square tests (χ^2^).

**Demographic factor**	**Melbourne zoo**	**Taronga zoo**	**Total**	***P*-value**
Number of participants	495 (78%)	143 (22%)	638	
**Residence**				
Living in Australia	392	82	474 (75%)	**<0.01**
Overseas	98	61	159 (25%)	
**Type of visitor**				
Zoo member	221	19	240 (38%)	**<0.01**
Non-zoo member	274	123	397 (62%)	
**Gender**				
Male	149	45	194 (31%)	0.81
Female	345	97	442 (69%)	
**Previously owned/Currently own a pet**				
Yes	463	121	584 (92%)	**<0.01**
No	32	21	53 (8%)	
**Age**				
18–25	119	30	149 (24%)	**<0.05**
26–35	144	48	192 (30%)	
36–45	125	23	148 (23%)	
46–55	31	18	49 (8%)	
55+	73	23	96 (15%)	
**Highest level of education**				
No formal school	0	1	1 (0.2%)	
Primary school	1	0	1 (0.2%)	
Secondary school	90	20	110 (17%)	0.56
Technical or further education institution (including TAFE College)	90	27	117 (18%)	
University or other higher education institution	309	91	400 (63%)	
Other education institution	5	2	7 (1.1%)	

*Melbourne zoo demographic information was obtained from Chiew et al. (2019b). P-values less than 0.05 are in bold*.

### Principal Component Analyses

A total of 35 attitudinal statements were subjected to PCAs with the question items, component loadings and Cronbach's α coefficients or Inter-item correlations given in Table 2. A total of 10 attitude components were extracted and found to be consistent at each zoo, with slight variation in the loadings (Table 2). Note that for Melbourne Zoo, “Positive visitor effect” and “Neutral visitor effect” components were obtained from Chiew et al. (2019b) while the remaining extracted attitudes components were obtained from new PCAs conducted in the present study to ensure consistency with the question items that loaded on the extracted components at Taronga Zoo. The labeling of the resulting components were similar to those found in Chiew et al. (2019b) (Table 2). Six individual question items including whether visitors thought little penguins are aggressive, timid, or cute and how visitors rated the welfare of the little penguins, the penguin enclosure and visitor experience at the enclosure were analyzed separately and not subjected to PCAs. This was because these items either, did not reliably measure the same underlying construct when subjected to PCA or used a different rating scale to the attitudinal statements (Table 3). These items were subjectively labeled as “Perceived Aggressiveness,” “Perceived Timidness,” “Perceived Cuteness,” “Welfare of the little penguins,” “Little penguin enclosure,” and “Visitor experience at the penguin enclosure” (Table 3).

**Table 2:** Extracted attitude components at each zoo after performing PCAs and, the corresponding question items on each component, their loadings and Cronbach's α coefficients.

**Extracted attitude component**	**Items**	**Melbourne Zoo (*****n*** **=** **495)**		**Taronga Zoo (*****n*** **=** **143)**	
		**Loadings**	**Cronbach's α**	**Loadings**	**Cronbach's α**
Positive penguin characteristics	Do you think little penguins are Playful?	0.86	0.77	0.81	0.64 (Inter-item correlation = 0.31)
	Do you think little penguins are Intelligent?	0.81		0.62	
	Do you think little penguins are Friendly?	0.75		0.63	
	Do you think little penguins are Social?	0.68		0.72	
Negative penguin welfare	Do you think the penguins are Frightened?	0.89	0.83	0.89	0.87
	Do you think the penguins are Stressed?	0.85		0.74	
	Do you think the penguins are Frustrated?	0.72		0.83	
	Do you think the penguins are Anxious?	0.68		0.80	
	Do you think the penguins are Subdued?	0.64		0.75	
	Do you think the penguins are Bored?	0.54		0.51	
Positive penguin welfare	Do you think the penguins are Alert?	0.89	0.78	0.88	0.74
	Do you think the penguins are Healthy?	0.82		0.65	
	Do you think the penguins are Happy?	0.75		0.72	
	Do you think the penguins are Expressing natural behaviors?	0.62		0.63	
	Do you think the penguins are Calm?	0.40		0.56	
Positive visitor effect	Do you think the penguins find visitors Entertaining?	0.89	0.78	0.92	0.86
	Do you think the penguins find visitors Interesting?	0.88		0.91	
	Do you think the penguins find visitors Novel?	0.72		0.82	
Neutral visitor effect	Do you think the penguins find visitors NOT fear-provoking?	0.86	0.59 (Inter-item correlation = 0.43)	0.81	0.47 (Inter-item correlation = 0.31)
	Do you think the penguins are unbothered by visitors?	0.82		0.81	
Positive enclosure characteristics	Do you think the penguin enclosure is Interesting to look at?	0.84	0.82	0.75	0.83
	Do you think the penguin enclosure is Well maintained?	0.83		0.84	
	Do you think the penguin enclosure is Natural looking?	0.83		0.71	
	The exhibit was engaging.	0.69		0.89	
Negative enclosure characteristics	Do you think the penguin enclosure is Restrictive?	0.92	0.79	0.92	0.87
	Do you think the penguin enclosure is Small?	0.88		0.94	
Experience with the penguins	I like being close to the penguins.	0.81	0.81	0.79	0.85
	It was exciting to see the little penguins.	0.81		0.87	
	I like seeing the penguins active and engaging in lots of behaviors.	0.77		0.79	
	It was entertaining to watch the little penguins.	0.75		0.86	
Learning	I learnt about a penguin's natural lifestyle.	0.90	0.88	0.92	0.89
	I learnt about penguin behavior when I was at the penguin exhibit.	0.89		0.90	
	I learnt about conservation issues related to penguins	0.87		0.85	
Visual barriers	Having one-way visual barriers where penguins cannot see visitors, but visitors can see penguins improves visitors' experience.	0.90	0.76	0.94	0.86
	Having one-way visual barriers where penguins cannot see visitors, but visitors can see penguins improves penguin welfare.	0.90		0.94	

*Inter-item correlations are also presented for components that had a Cronbach's α below the criterion (i.e., α < 0.70)*.

**Table 3:** Differences between Melbourne Zoo and Taronga Zoo scale mean scores and rating questions (± standard error of the mean, SEM) identified from univariate analyses.

	**Overall**	**Melbourne Zoo**	**Taronga Zoo**	***P*-value**
**Scale mean scores (Likert scale 1–5: 1** **=** **strongly disagree, 5** **=** **strongly agree)**				
Positive penguin characteristics	3.88 ± 0.04	3.89 ± 0.03	3.88 ± 0.07	0.99
Perceived Aggressiveness	1.95 ± 0.06	1.79 ± 0.04	2.11 ± 0.11	**<0.01**
Perceived Timidness	3.32 ± 0.06	3.55 ± 0.05	3.10 ± 0.11	**<0.01**
Perceived Cuteness	4.55 ± 0.05	4.47 ± 0.04	4.63 ±0.09	0.09
Negative penguin welfare	2.47 ± 0.04	2.59 ± 0.03	2.36 ± 0.08	**<0.01**
Positive penguin welfare	3.74 ± 0.04	3.71 ± 0.03	3.77 ± 0.07	0.40
Positive visitor effects	2.82 ± 0.05	2.79 ± 0.04	2.85 ± 0.09	0.54
Neutral visitor effects	3.30 ± 0.05	3.23 ± 0.04	3.36 ± 0.10	0.20
Positive enclosure characteristics	3.57 ± 0.05	3.49 ± 0.04	3.65 ± 0.10	0.13
Negative enclosure characteristics	2.99 ± 0.06	2.99 ± 0.05	2.99 ± 0.12	0.97
Experience with the penguins	4.13 ± 0.06	3.99 ± 0.03	4.27 ± 0.08	**<0.01**
Learning	3.04 ± 0.06	2.78 ± 0.05	3.30 ± 0.12	**<0.01**
Visual barriers	3.53 ± 0.06	3.67 ± 0.04	3.40 ± 0.10	**<0.05**
**Rating questions (scale 1–10: 1** **=** **very poor, 10** **=** **excellent)**				
Welfare of the little penguins	7.68 ± 0.12	7.59 ± 0.09	7.77 ± 0.22	0.44
Little penguin enclosure	7.02 ± 0.14	6.97 ± 0.11	7.07 ± 0.26	0.73
Visitor experience at the penguin enclosure	6.96 ± 0.15	6.55 ± 0.11	7.37 ± 0.27	**<0.01**

*P-values less than 0.05 and 0.01 are in bold*.

### Differences in Visitor Attitudes Between Zoos

MANOVAs were performed to examine the effect of the demographic variables on visitor attitudes. A total of 16 variables to represent visitor attitudes (i.e., the 10 extracted attitude components from PCAs and six individual question items) were used as the dependent variables. The independent variables were the demographics variables: “Residence,” “Type of visitor,” “Gender,” “Pet ownership,” “Age,” and “Education.” Few effects of the demographic variables on visitor attitudes were found. A significant effect of “Pet ownership” on the “Perceived Cuteness” of little penguins, “Experience with the penguins” and “Visual Barriers” was found [*F*_(16, 417)_ = 1.98, *p* < 0.05, Wilk's Lambda = 0.93, partial η^2^ = 0.07]. People who were had previously or currently owned a pet had more positive attitudes that little penguins are cute [*F*_(1, 432)_ = 11.15, *p* < 0.05, partial η^2^ = 0.03] and positive attitudes toward “Experience with the penguins” [*F*_(1, 432)_ = 5.82, *p* < 0.01, partial η^2^ = 0.01] and “Visual Barriers” [*F*_(1, 432)_ = 5.50, *p* < 0.05, partial η^2^ = 0.01] compared to visitors that did not previously or currently own a pet. No interactions were found between any of the demographic variables and zoo property on visitor attitudes (*p* > 0.05).

Table 3 presents the scale mean scores of the 16 attitudes variables, where the higher the scale mean score, the more agreement and therefore, more positive attitude. Based on the scale mean scores, overall, visitors at both zoos had positive attitudes toward penguin characteristics, their welfare and visitor experience, neutral attitudes toward positive and neutral effects of visitors on little penguins and relatively positive attitudes toward the penguin enclosure and visual barriers (Table 3). When the effect of zoo property (independent variable) on the 16 attitude variables (dependent variables) was examined by performing a MANOVA, it was found that there were some significant differences in visitor attitudes between the two zoos [*F*_(16, 419)_ = 4.37, *p* < 0.01, Wilk's Lambda = 0.86, partial η^2^ = 0.14]. To identify which visitor attitudes differed between the two zoos, the results for the 16 attitudes variables were considered separately through univariate analyses (Table 3). Visitors at both zoos tended to disagree that the penguins were frightened, stressed, frustrated, anxious, subdued, or bored (PCA component “Negative penguin welfare”) but, there was a slightly higher level of agreement at Melbourne Zoo [*F*_(1, 434)_ = 7.56, *p* < 0.01, partial η^2^ = 0.02]. Also, visitors at both zoos reported positive “Experience with the penguins” however, there were greater positive experiences at Taronga Zoo compared to Melbourne Zoo [*F*_(1, 434)_ = 11.76, *p* < 0.01, partial η^2^ = 0.03]. This was also reflected in the overall visitor experience at the penguin enclosure, which was rated higher at Taronga Zoo compared to Melbourne Zoo [*F*_(1, 434)_ = 8.11, *p* < 0.01, partial η^2^ = 0.02]. For both zoos, visitors had a fairly neutral response toward “Learning” where visitors neither agreed nor disagreed that they learnt about a penguin's natural lifestyle, penguin behavior and conservation issues related to penguins while at the penguin exhibit. However, visitors at Melbourne Zoo tended to disagree that they learnt about the penguins to a greater extent than did visitors at Taronga Zoo [*F*_(1, 434)_ = 16.04, *p* < 0.01, partial η^2^ = 0.04]. Also, visitors at Melbourne Zoo had more positive attitudes toward “Visual barriers” compared to visitors at Taronga Zoo [*F*_(1, 434)_ = 6.00, *p* < 0.05, partial η^2^ = 0.01]. There were also some significant differences in visitors' responses to individual questionnaire items. “Perceived Aggressiveness” of little penguins was higher at Taronga Zoo [*F*_(1, 434)_ = 7.89, *p* < 0.01, partial η^2^ = 0.02] although at both zoos, visitors tended overall to disagree that little penguins are aggressive. “Perceived Timidness” of little penguins was greater at Melbourne Zoo [*F*_(1, 434)_ = 13.27, *p* < 0.01, partial η^2^ = 0.03] although at both zoos, visitor tended to agree overall that little penguins are timid. Note that, partial η^2^ is a measure of effect size and the values ranged from small (i.e., 0.01) to medium (i.e., 0.06) and one that was large (i.e., 0.14) (Cohen, 1988).

### Relationship Between Visitor Attitudes and How Visitors Rated Little Penguin Welfare, the Enclosure, and Visitor Experience

When zoo property was controlled for, visitor attitudes were found to be significantly correlated with how visitors rated little penguin welfare, the enclosure and visitor experience at the penguin enclosure (*p* < 0.05; Table 4). Visitor attitudes toward “Positive penguin characteristics,” “Positive penguin welfare,” positive and neutral visitor effects, “Positive enclosure characteristics,” “Experience with the penguins,” and “Learning” were positively correlated with higher ratings of little penguin welfare, the enclosure and visitor experience (Table 4). While, visitor attitudes toward “Negative penguin welfare”, “Negative enclosure characteristics” and “Visual barriers” were negatively associated with how visitors rated little penguin welfare, the enclosure and visitor experience (Table 4). Partial correlations also showed that the individual question items related to little penguin aggressiveness, timidness, and cuteness were associated with how visitors rated little penguin welfare, the enclosure and visitor experience. “Perceived Aggressiveness” and “Perceived Timidness” of little penguins were negatively correlated with how visitors rated little penguin welfare whereas “Perceived Cuteness” was positively associated with how visitors rated their experience at the penguin enclosure (Table 4). Note that, with the exception of “Perceived timidness,” “Perceived cuteness,” and “Visual barriers,” correlations were within the strong correlation range, 0.60–0.79 and moderate range, 0.40–0.59, and a few in the weak range, 0.20–0.39 (Evans, 1996).

**Table 4:** Partial correlations between visitor attitudes and how visitors rated little penguin welfare, the enclosure and visitor experience.

	**Welfare of the little penguins**	**Little penguin enclosure**	**Visitor experience at the penguin enclosure**
Positive penguin characteristics	0.14**	0.21**	0.28**
Perceived Aggressiveness	−0.12**	−0.12**	−0.17**
Perceived Timidness	−0.10^*^	−0.07	−0.05
Perceived Cuteness	0.06	0.08	0.18**
Negative penguin welfare	−0.43**	−0.44**	−0.44**
Positive penguin welfare	0.54**	0.55**	0.59**
Positive visitor effects	0.27**	0.29**	0.26**
Neutral visitor effects	0.29**	0.27**	0.26**
Positive enclosure characteristics	0.47**	0.70**	0.66**
Negative enclosure characteristics	−0.44**	−0.60**	−0.46**
Experience with the penguins	0.34**	0.38**	0.46**
Learning	0.34**	0.43**	0.52**
Visual barriers	−0.09^*^	−0.15**	−0.10^*^

* *Correlation is significant at the 0.05 level (2-tailed)*.

** *Correlation is significant at the 0.01 level (2-tailed)*.

## Discussion

The same attitude components were extracted at both zoos which suggests that the attitude dimensions of visitors were stable between the two zoos. This indicates that these measures of attitudes were stable over time and location. In other words, it suggests that visitors at the two zoos evaluated little penguins, penguin welfare, visitor effects, the enclosure, learning, visual barriers, and visitor experience in a similar manner, resulting in similar visitor attitudes, or more specifically visitor beliefs. This is the cognitive component of attitudes that was assessed in the present study to infer the attitudes of visitors. Overall, visitors at both zoos had positive attitudes toward little penguins, evident through the positive visitor beliefs that little penguins are playful, intelligent, friendly and social and were in a positive welfare state. Visitors also generally had neutral attitudes toward positive and neutral effects of visitors on little penguins and learning, and relatively positive attitudes toward the penguin enclosure and visual barriers. We also found that the more positive visitor attitudes were toward penguins, their welfare, the enclosure, experience with the penguins and learning, the higher visitor ratings were for penguin welfare, the enclosure and visitor experience. Our results aligns with previous research that also indicates zoo visitors have positive attitudes toward zoo animals and their enclosures when zoos provide positive experiences for visitors (Woods, 2002; Clayton et al., 2009; Miller et al., 2013; Yilmaz et al., 2017). Consequently, the present study results are encouraging for both zoos as it suggests the two zoos are providing positive experiences for visitors at the penguin exhibit that may be related to positive visitor attitudes which is important for the overall ethical foundation of zoos (Maple and Perdue, 2013; Sherwen et al., 2018). However, despite these positive attitudes, there is evidence at both zoos that close visitor contact increases avoidance of visitors in little penguins (Chiew et al., 2019a, 2020).

Visitor attitudes were surveyed in conjunction with our experiments that investigated the effects of regulating visitor contact on little penguin behavior at Melbourne and Taronga Zoo (Chiew et al., 2019a, 2020). Visitor contact was regulated by making alterations or manipulations to the exhibit which included, a physical barrier placed 2 m from the enclosure at Melbourne Zoo and a visual barrier that obstructed the view between visitors and penguins at a single viewing window at Taronga Zoo (Chiew et al., 2019a, 2020). Chiew et al. (2019a) found that the close viewing proximity of visitors can be fear-provoking for little penguins at Melbourne Zoo, shown by the increased number of penguins huddling, vigilant, avoiding the visitor viewing area and decreased number of penguins surface swimming. The close visitor viewing proximity also increased intense visitor behaviors including looming over the pool ledge, sudden movement and tactile contact with the pool's water. In comparison, little penguins at Taronga Zoo showed some avoidance of the main visitor viewing window when visitor contact was not obstructed, indicated by the reduced presence, reduced preening in the water and increased vigilance by little penguins in the area near this main window (Chiew et al., 2020). The unobstructed window also allowed visitors to view the penguins up close and engage in potentially fear-provoking behaviors such as banging on the window, loud vocalizations, and sudden movement at this window (Chiew et al., 2020). Despite the intense and potentially fear-provoking visitor behaviors observed at the two zoos which were likely perceived by little penguins as threatening, visitors had positive attitudes toward little penguins, their welfare, the enclosure, experience with the penguins and visual barriers. Visitors also overall rated their experience at the enclosure relatively high. This suggests that the positive visitor attitudes and experience found in the present study, may be increasing these intense and potentially negative behaviors in visitors toward penguins which contrasts with the research on stockperson-animal relationships and the handful of studies that have found positive visitor attitudes toward wildlife may reduce intense and harmful behaviors toward wildlife (Blaney and Wells, 2004; Barney et al., 2005; Hemsworth and Coleman, 2011; Pearson et al., 2014b; Munoz et al., 2019). It is possible that the positive visitor attitudes and positive visitor experience found in the present study may have increased visitors' desire to interact or be in close contact with penguins. Thus, this may have motivated visitors to engage in behaviors to seek the penguins' attention which visitors may not be aware may be perceived as threatening for the penguins (Chiew et al., 2019b). The potential lack of awareness of visitor effects on little penguins is consistent with the neutral visitor attitudes toward positive and neutral visitor effects found in the present study (i.e., neither agreed nor disagreed). This could be addressed by effective communication and educational strategies to raise visitor awareness about visitor effects or alter visitor attitudes and perceptions of interacting with zoo animals (Chiew et al., 2019b; Learmonth, 2020). However, we were not able to directly correlate the attitudes of each visitor with their behavior and did not examine visitors' attitudes specifically toward the behaviors that they, as visitors, engage in toward little penguins. Consequently, further research on visitor attitudes and their link with visitor behavior toward zoo animals is still required.

Even though the overall attitudes of visitors at both zoos were positive, there were some differences in visitor attitudes between the zoos. The visitor attitudes that differed between the two zoos were attitudes toward the perceived “aggressiveness” and “timidness” of little penguins, “negative penguin welfare,” “experience with the penguins,” “learning,” “visual barriers” and the way visitors rated their overall experience at the penguin enclosure. Why only these seven, out of 16, attitudes specifically differed is unclear but suggests that visitors at the two zoos likely had different perceptions of the enclosures, behavior of the little penguins and their experience at the enclosure. However, it is well-understood that attitudes toward animals can vary as a result of variation in demographic, socioeconomic and cultural factors as well as past experiences with animals (Bjerke et al., 2003; Taylor and Signal, 2005; Prokop and Tunnicliffe, 2010; Clucas and Marzluff, 2012). Some of these factors may explain some of the differences in visitor attitudes and visitor experience between the zoos in the present study. There were some slight differences in visitor demographics at the two zoos where participants that completed the questionnaire at Melbourne Zoo were primarily locals living in Australia, were balanced between zoo members and non-zoo members, had a greater proportion of pet owners or people who had previously owned a pet and participants aged between 26–35 and 36–45 years old compared to participants at Taronga Zoo. These differences in demographics may have been responsible for the differences in visitor attitudes and experience between the zoos. However, in the MANOVAs, no interactions between the demographic variables and zoo property were found which suggests the differences in visitor attitudes and visitor experience were not attributable to the differences between the two zoos in visitor demographics.

As previously mentioned, visitors in the present study were surveyed in conjunction with our studies that examined the effects of regulating visitor contact on little penguin behavior by using exhibit manipulations which may have affected the differences in visitor attitudes and experience between the two zoos. Indeed, Chiew et al. (2019b) found that regulating visitor-penguin interactions using a physical barrier placed 2 m from the penguin exhibit and signs, affected some visitor attitudes at Melbourne Zoo, particularly attitudes toward positive penguin characteristics, neutral visitor effects and physical barriers. However, the physical barrier and signs had no effect on other visitor attitudes such as attitudes toward penguin welfare, the enclosure, visitor experience and the way visitors rated these items. This suggests there was no detrimental impact on the overall attitudes toward little penguins, the enclosure and visitor experience when using a physical barrier and signs to regulate visitor-penguin interactions (Chiew et al., 2019b). While not specifically examined in the present study, exploratory analyses found no interaction between having a barrier present (physical or visual) and zoo property. This suggests the differences in visitor attitudes and experience between the two zoos are unlikely to have been influenced by the exhibit manipulations at each zoo (Chiew et al., 2019b). Interestingly, more positive attitudes toward visual barriers were found at Melbourne Zoo compared to visitor attitudes at Taronga Zoo. This may be because no visual barriers were used to regulate visitor-penguin interactions at Melbourne Zoo and no detrimental impact of a physical barrier or signs was found on visitor experience (Chiew et al., 2019a,b). In comparison, a visual barrier was used at Taronga Zoo which reduced the view of penguins at a single visitor viewing window (Chiew et al., 2020).

The exhibit manipulations at the two zoos may not have influenced the differences in visitor attitudes and experience between the zoos *per se*, but the changes in zoo animal behavior as a result of these manipulations may have. For example, camouflage netting installed onto the viewing area of the exhibit was found to reduce conspecific-directed aggression and stereotypic behavior in gorillas and improved visitor perceptions of gorillas where they were perceived as more exciting and less aggressive (Blaney and Wells, 2004). The improvement in visitor perceptions may not have been only because of the presence of the camouflage netting but also the change in gorilla behavior as a result of the netting. Consequently, the behavior of penguins at the two zoos may be a factor that influenced the differences in visitor attitudes and experience between the zoos. Several studies have shown animal behavior can influence the attitude of visitors and visitor experience. Zoo animals that are the active and behaviorally diverse are associated with increased positive visitor perceptions of animals, visitor experience and increased self-reported positive affective responses and conservation intent in visitors (Anderson et al., 2003; Godinez et al., 2013; Hacker and Miller, 2016; Luebke et al., 2016). Recently, Chiew et al. (2019b) also showed that little penguin behavior is correlated with visitor attitudes. When little penguins at Melbourne Zoo were more visible, active (e.g., swimming and diving) and close to the visitor viewing area, visitors had more positive attitudes toward little penguins, penguin welfare, visitor effects, the enclosure, learning, experience and exhibit manipulations and, rated higher the welfare of the penguins, the enclosure and visitor experience at the penguin exhibit (Chiew et al., 2019b). Consequently, in the present study, the behavior of little penguins at each zoo may have influenced the differences in visitor attitudes and experience between the zoos. Further analyses to examine the relationship between penguin behavior and visitor attitudes particularly at Taronga Zoo needs to be conducted to determine if similar relationships to those found at Melbourne Zoo exist. Nevertheless, it is more likely that these differences in visitor attitudes and experience between the zoos are the result of the intrinsic differences in enclosure design and the subsequent variation in visitor-penguin interactions at the two zoos.

Both zoos differed quite dramatically in the design of their penguin enclosures which influenced the type of interactions that were possible between the visitors and little penguins. At Melbourne Zoo, the design of the enclosure allowed visitors to view the penguins from three sides of the enclosure, with the main length of the pool being the common viewing area and where there were opportunities, if visitors chose, to make tactile contact with the pool by looming over the pool edge (Chiew et al., 2019a). Chiew et al. (2019a) found that the close viewing proximity of visitors had a pronounced effect on little penguin fear responses to visitors at Melbourne Zoo. But penguin fear responses were reduced when visitors were further away from the enclosure by using a physical barrier (Chiew et al., 2019a). At Taronga Zoo, the penguin enclosure was long and narrow, had a long watercourse and elongated stretch of land where the visitor viewing area was limited to one side of the enclosure, positioning visitors below penguins and allowed visitors to primarily view penguins underwater through tall glass windows (Chiew et al., 2020). As previously described, there was evidence of some avoidance of visitors by little penguins when visitor contact was not obstructed by a visual barrier at Taronga Zoo. This suggests that visitor contact also had an effect on little penguin fear responses at Taronga Zoo. But in comparison to the penguins at Melbourne Zoo, the penguin fear responses at Taronga Zoo was less pronounced because of the design of the Taronga Zoo penguin enclosure that helped minimize visitor contact by restricting the viewing area to one side of the enclosure and provided a retreat area where penguins were visually hidden from visitors. Consequently, it is possible that these differences in enclosure design and visitor-penguin interactions at the two zoos may explain some of the differences in visitor attitudes and visitor experience between the zoos. Studies that have examined the effects of enclosures on zoo visitors has primarily focused on the effects of naturalistic vs. non-naturalistic enclosures (Rhoads and Goldsworthy, 1979; Finlay et al., 1988; Melfi et al., 2004; Nakamichi, 2007; Mun et al., 2013; Luebke et al., 2016). But research has yet to examine the effects of other characteristics of enclosures on visitors such as the position of visitors relative to the animals while viewing animals, restriction of the visitor viewing area to one side vs. all sides of an enclosure or the presence or absence of glass viewing windows. Further examination of the specific exhibit design characteristics that may be influencing the positive, neutral, and negative visitor attitudes observed in the present study is recommended.

It should be noted that the magnitudes of the significant differences in attitudes (i.e., the scale mean scores) observed in the present study are numerically small, ranging from 0.23 to 0.52 (for the 5 point Likert scale variables) and differed by about 0.80 for visitor experience ratings (out of 10) at the enclosure. Furthermore, the effect sizes (partial η^2^) mostly ranged from small to medium (i.e., 0.01 to 0.06). However, it is unclear what the practical implications of our results are because it is not possible to interpret the absolute magnitude of these measures. Comparisons would need to be made between a larger number of zoos or enclosures that are more markedly different than the penguin enclosures at the two zoos studied here to permit a better understanding of the basis for the differences in attitudes. This is because visitors at each zoo appeared to view the enclosures similarly shown by the enclosures being rated positively at both zoos, scoring ~7/10. Yet, the penguin enclosures at the two zoos did differ in design, especially in terms of how visitors were able to see and interact with the penguins. At Taronga Zoo, visitors viewed penguins up close while the penguins were in the pool compared to Melbourne Zoo where visitors primarily viewed penguins from afar because the penguins were mostly withdrawn on land. Consequently, further investigation to understand what animal and enclosure characteristics are important to visitors that may be similar across zoos is needed to determine the practical implications of these differences in visitor attitudes.

While the results from the present study cannot specifically be extrapolated to other zoos, it does provide an approach that can be extrapolated to examine visitor attitudes in conjunction with the effect of visitors on zoo animals. This type of approach has yet to be extended to other zoo species but is a promising avenue to help better manage visitor-animal interactions by understanding both sides of the zoo visitor-animal relationship. Our results also provide valuable insight into visitor attitudes toward an understudied zoo species, little penguins, and visitor experience at two Australian zoos and, a foundation for future research to build upon which can be applied at other zoos. In particular, our study has highlighted the need for further examination of the relationship between visitor attitudes and the behavior of visitors toward zoo animals and how visitors assess zoo animal welfare, animal enclosures and their experience at exhibits. These suggestions for future research are important because firstly, human behavior can significantly affect animal welfare (Hemsworth et al., 2018; Sherwen and Hemsworth, 2019). Therefore, understanding the attitudes that may influence human behavior toward animals can help develop human behavior change interventions to continuously improve the lives of animals not only in zoos but across the different animal industries (Coleman, 2010; Hemsworth et al., 2018; Glanville et al., 2020). Secondly, the present study found ratings for penguin welfare, the enclosure and visitor experience were only moderately high and attitudes toward learning were fairly neutral at both zoos. A number of studies have found that visitors assess animal welfare based on aesthetics like enclosure style and the expression of active behaviors (e.g., play, climbing, and eating behaviors) where the more naturalistic an enclosure looks and active an animal is, the better visitors perceived the welfare of the animal (Rhoads and Goldsworthy, 1979; Finlay et al., 1988; Melfi et al., 2004; Nakamichi, 2007; Luebke et al., 2016; Chiew et al., 2019b). While visitors are not specifically qualified to make robust assessments of animal welfare or other aspects of the zoo, understanding how visitors rate zoo animal welfare, enclosures and their experience and what visitors are learning while at zoos is still valuable. Through this type of information, zoos can identify areas of improvement and develop strategies, for example, to enable visitors to make more informed judgements about animal welfare and zoo enclosure design or enhance visitor knowledge of the potential effects visitors can have on zoo animals (Melfi et al., 2004; Coleman, 2010).

## Conclusions

This study is the first study, to our knowledge, that compared the attitudes of visitors, by assessing the cognitive (belief) component of attitudes, toward zoo-housed little penguins, their enclosure and visitor experience at two Australian zoos. There were some differences in visitor attitudes and visitor experience between the two zoos. While the reasons for these differences were not clear, they did not appear to be due to visitor demographics or the exhibit manipulations imposed at each zoo. It is possible that the differences may be attributable to the enclosures themselves which differed in their design and influenced the way penguins behaved and how visitor interacted with the penguins, despite both enclosures were perceived relatively positively at both zoos. Nevertheless, this study found visitors at both zoos, overall, had positive attitudes toward the little penguins, penguin welfare, the enclosure and visitor experience which was related to higher visitor ratings of the welfare of the penguins, enclosure and visitor experience at the enclosure. But it remains unclear how these positive attitudes and positive visitor experience may be influencing the way visitors behave toward zoo-housed little penguins. Results from this study have increased our understanding of visitor attitudes toward little penguins and visitor experience at two Australian zoos and provides a foundation for future research to build upon.

## Data Availability Statement

The datasets generated for this study are available on request to the corresponding author.

## Ethics Statement

The studies involving human participants were reviewed and approved by the Veterinary and Agricultural Sciences Human Ethics Advisory Group, Faculty of Veterinary and Agricultural Sciences, University of Melbourne, Australia (Ethics Application 1545739.1 and 1545739.3). The patients/participants provided their written informed consent to participate in this study.

## Author Contributions

SC, PH, SS, VM, AB, and GC designed the study. GC, SS, and AB assisted with focus groups at Melbourne Zoo and Taronga Zoo, respectively. SC, with assistance from SS and AB, liaised with the keepers and other zoo staff at both Melbourne and Taronga Zoo. SC organized and carried out the data collection with the help of student volunteers, interns and research assistant from the Animal Welfare Science Centre (University of Melbourne, Australia), who were also organized and trained by SC. SC collated all data and with the aid of GC, performed the statistical analyses of the data and interpretation. SC wrote the paper. GC, PH, VM, SS, and AB provided feedback and additions to the paper. All authors contributed to the article and approved the submitted version.

## Conflict of Interest

The authors declare that the research was conducted in the absence of any commercial or financial relationships that could be construed as a potential conflict of interest.
